# Healthcare utilization and costs among high-need and frail Mexican American Medicare beneficiaries

**DOI:** 10.1371/journal.pone.0262079

**Published:** 2022-01-14

**Authors:** Maricruz Rivera-Hernandez, Amit Kumar, Lin-Na Chou, Tamra Keeney, Nasim Ferdows, Amol Karmarkar, Kyriakos S. Markides, Kenneth Ottenbacher

**Affiliations:** 1 Department of Health Services, Policy & Practice, Brown University School of Public Health, Providence, Rhode Island, United States of America; 2 Center for Gerontology and Healthcare Research, Brown University School of Public Health, Providence, Rhode Island, United States of America; 3 College of Health and Human Services, Northern Arizona University, Flagstaff, Arizona, United States of America; 4 Center for Health Equity Research, Northern Arizona University, Flagstaff, Arizona, United States of America; 5 Office of Biostatistics, University of Texas Medical Branch, Galveston, Texas, United States of America; 6 Department of Preventive Medicine and Population Health, University of Texas Medical Branch, Galveston, Texas, United States of America; 7 Department of Health Administration and Policy, Hudson College of Public Health, The University of Oklahoma Health Sciences Center, Oklahoma City, OK, United States of America; 8 Department of Physical Medicine and Rehabilitation, Virginia Commonwealth University, Richmond, Virginia, United States of America; 9 Sheltering Arms Institute, Richmond, Virginia, United States of America; 10 Sealy Center on Aging, University of Texas Medical Branch, Galveston, Texas, United States of America; 11 Division of Rehabilitation Sciences, School of Health Professions, University of Texas Medical Branch, Galveston, Texas, United States of America; University of Utah, UNITED STATES

## Abstract

**Objectives:**

To examine Medicare health care spending and health services utilization among high-need population segments in older Mexican Americans, and to examine the association of frailty on health care spending and utilization.

**Methods:**

Retrospective cohort study of the innovative linkage of Medicare data with the Hispanic Established Populations for the Epidemiologic Study of the Elderly (H-EPESE) were used. There were 863 participants, which contributed 1,629 person years of information. Frailty, cognition, and social risk factors were identified from the H-EPESE, and chronic conditions were identified from the Medicare file. The Cost and Use file was used to calculate four categories of Medicare spending on: hospital services, physician services, post-acute care services, and other services. Generalized estimating equations (GEE) with a log link gamma distribution and first order autoregressive, correlation matrix was used to estimate cost ratios (CR) of population segments, and GEE with a logit link binomial distribution was applied to estimate odds ratios (OR) of healthcare use.

**Results:**

Participants in the major complex chronic illness segment who were also pre-frail or frail had higher total costs and utilization compared to the healthy segment. The CR for total Medicare spending was 3.05 (95% CI, 2.48–3.75). Similarly, this group had higher odds of being classified in the high-cost category 5.86 (95% CI, 3.35–10.25), nursing home care utilization 11.32 (95% CI, 3.88–33.02), hospitalizations 4.12 (95% CI, 2.88–5.90) and emergency room admissions 4.24 (95% CI, 3.04–5.91).

**Discussion:**

Our findings highlight that frailty assessment is an important consideration when identifying high-need and high-cost patients.

## Introduction

Serving older adults with complex healthcare needs requires an improved understanding of how management of chronic conditions is influenced by frailty and social risk factors [1]. In the United States, a small proportion of older adults account for a staggering amount of Medicare spending [2]. Thus, significant efforts have been devoted to identifying beneficiaries who are high-need, high-cost (HNHC) [3–6]. Prior literature has revealed that a large portion of HNHC individuals have multiple chronic conditions and are from minority backgrounds, including Hispanic older adults [5,7]. However, there are two limitations related to these investigations. First, prior studies have not included other potential predictors that may contribute to being HNHC (e.g., frailty, cognitive impairment, and social risk factors). Second, there is no information regarding HNHC status among Hispanic subgroups (e.g., Mexican American beneficiaries).

Hispanic beneficiaries are more likely than non-Hispanic white beneficiaries to have low-incomes and be dually-eligible for Medicare and Medicaid [8]. For instance, in 2019, about 50% of Medicare beneficiaries had annual incomes close to $30,000, compared to $14,000 for Hispanics [9]. High levels of poverty among Hispanic subgroups suggest that out-of-pocket costs may be a burden for older adults, including those with disabilities. However, there is limited knowledge about Medicare utilization and spending among Hispanic subgroups (e.g., Mexican Americans or Puerto Ricans). Hispanics are the largest ethnic minority group in the U.S. (about 18% of the population) [10] and have a higher prevalence of multiple chronic conditions, functional limitations, frailty and cognitive impairment [11]. However, heterogeneity among Hispanic sub-groups represented by differences in life expectancy, number of chronic conditions, and variations in health services utilization results in a wide range of health care spending [12]. For example, people of Mexican origin account for over 60% of the US Hispanic population and may live longer with multiple chronic conditions and disability or frailty [12,13].

The race and ethnicity variable in the Master Beneficiary Summary File, part of the Medicare datasets, does not contain information regarding Hispanic origin categories (e.g., Puerto Rican, Mexican, Cuban). Thus, in order to understand healthcare cost and utilization within Hispanic subgroups we need to employ innovative data linkages using survey data and Medicare claims. The purpose of this study was to examine health services utilization and spending across previously defined high-cost population segments and evaluate the association of frailty on health care spending/utilization among those with high-cost and high-need among older Mexican Americans.

### Conceptual framework

Joynt and colleagues [14] proposed an approach to identify high-cost patients (using Medicare claims) that identified beneficiaries as having disability, frailty, and via the number of chronic conditions. Then, they characterized spending among the different subpopulations (inpatient, outpatient, post-acute care, other). We adapted their approach to classify Mexican American beneficiaries into four mutually exclusive high-cost segments on the basis of multimorbidity: major complex chronic illness, defined as those with at least 3 complex chronic conditions or at least 6 other chronic conditions; minor complex chronic illness, defined as those with 1 or 2 complex chronic conditions and with 1–5 other chronic conditions; simple chronic illness, defined as those without complex chronic conditions and with 1–5 other chronic conditions; and comparatively healthy (See Supporting Information S1 Table for this classification based on the chronic conditions in the MBSF). Due to the small number of people that were relatively healthy, this last category was regrouped with the simple chronic illness group for a total of three segments. Distinct from this proposed approach by Joynt and colleagues [14], we separately examined the potential contribution of frailty status to outcomes across high-cost segments. Our rationale for this is that frailty is a commonly observed geriatric syndrome that is highly prevalent among older adults with multiple chronic conditions [15] and is independently associated with poor health outcomes and high healthcare utilization [16–20].

## Methods

### Data source and study population

We used data from the Hispanic Established Populations for the Epidemiologic Study of the Elderly (H-EPESE) linked with the Centers for Medicare and Medicaid Services (CMS) Medicare Beneficiary Summary File (MBSF) and the MBSF: Cost and Utilization Segment. The H-EPESE is a longitudinal population-based study representative of Mexican Americans aged 65 years and older residing across the southwestern region of the U.S., Texas, New Mexico, Colorado, Arizona, and California [21]. The H-EPESE employed an area-probability sample that used counties in the Southwestern states with the higher numbers of older Mexican Americans and included respondents who were born in or outside of the US. The current study included Medicare data from the years 2000–2013.

**Fig 1:**
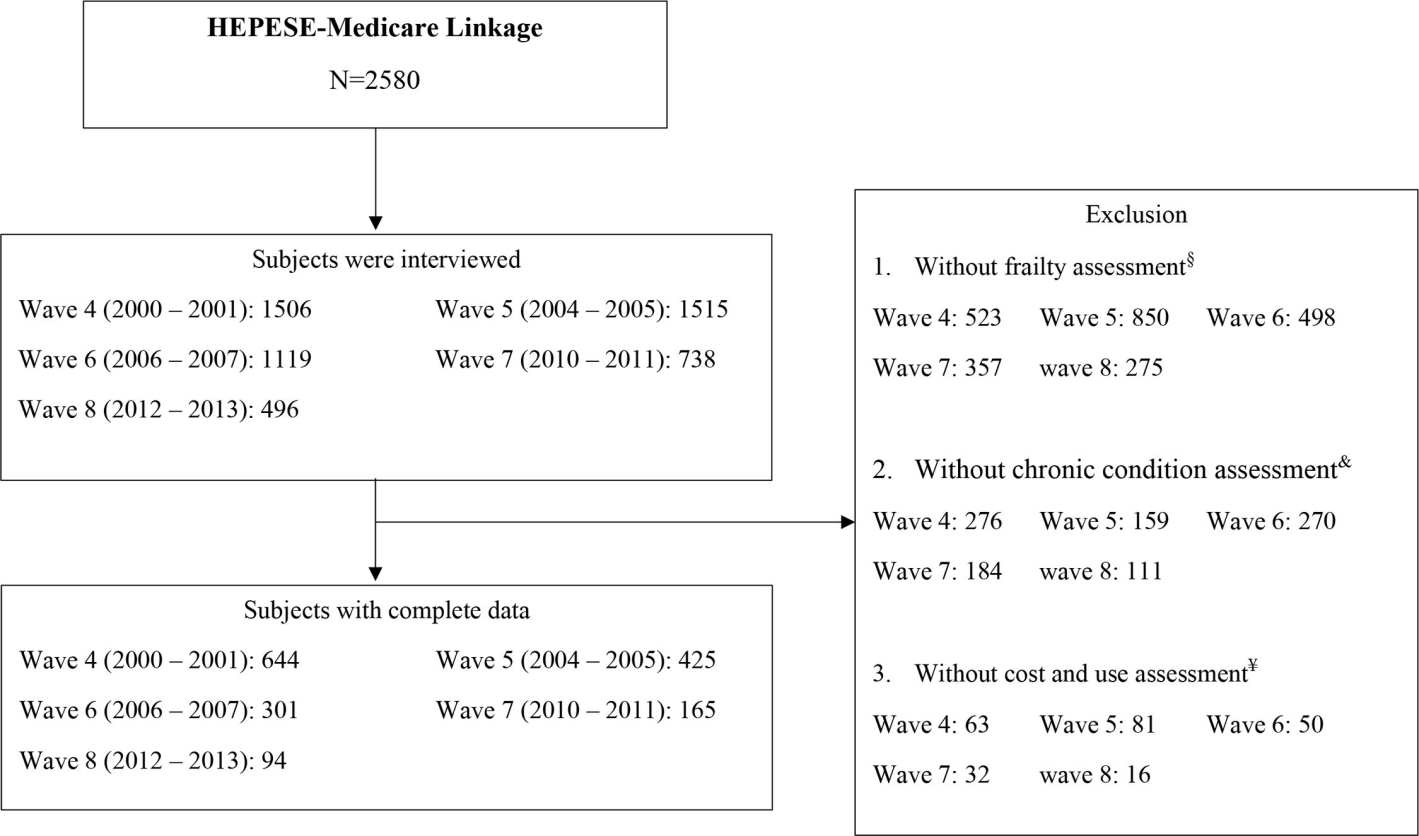
Flowchart of cohort selection. § Modified five criteria frailty assessment. & The Chronic Conditions (CC) segment of the Master Beneficiary Summary File (MBSF) was used to define chronic conditions; MBSF-CC files in 2001, 2005, 2007, 2011, and 2013 were used for wave 4, 5, 6, 7 and 8 separately; a list of the 37 conditions included in the study is presented in the Supporting Information S1 Table. ¥ The cost and use segment of MBSF files in 2002, 2006, 2008, 2012, and 2014 were used for wave 4, 5, 6, 7 and 8 separately.

Among the HEPESE-Medicare population (N = 2,580) we included the following: participants with complete frailty assessment, Medicare chronic conditions flags, and Fee-for-Service (FFS) enrollment from waves 4 (2000–2001), 5 (2004–2005), 6 (2006–2007), 7 (2010–2011) or 8 (2012–2013) (See Fig 1). We used the Chronic Conditions (CC) segment of the Master Beneficiary Summary File (MBSF) to identify chronic conditions (a list of the 37 conditions included in the analysis is presented in S1 Table). MBSF-CC files in 2001, 2005, 2007, 2011, and 2013 were used for wave 4, 5, 6, 7 and 8. This captured chronic conditions closer to the time of the survey interview. The MBSF: Cost and Utilization Segment used years from 2002, 2006, 2008, 2012, and 2014 for wave 4, 5, 6, 7 and 8. This captured healthcare utilization after the survey interview. Each participant contributed one to five years of Medicare spending and use of services. As a result, there were 863 participants that contributed 1,629 person years of information.

#### Ethics statement

Data were reviewed and approved by the University of Texas Medical Branch review board. A data use agreement was established with CMS. The H-EPESE data were collected via in-home interviews and informed consent was obtained prior to participation in the study. Detailed information about the survey design and data collection can be found in the H-EPESE documentation [22]. The data included a total of nine waves, collected from 1993 to 2017. Publicly available data are maintained by the *National Archive of Computerized Data on Aging* (NACDA) which is part of the Inter-university Consortium for Political and Social Research (ICPSR) [23]. Public files are available to anyone and do not require an ICPSR membership [24].

We used a deterministic method to link H-EPESE data with Medicare claims. The process was based on unique identifiers based on Social Security numbers, date of birth, and sex. This information was submitted to CMS (3,291 H-EPESE participants), which generated a list of 3,175 beneficiary IDs, 2,650 of whom were alive on or before December 31, 1998. There were 70 participants eliminated due to inconsistent records of sex, birth date, date of death, or county of residence, resulting in an eligible sample of 2,580. The methodology related to this linkage has been explained in detail in prior studies [25,26].

#### Outcome variables

*Medicare spending*. First, Medicare spending per beneficiary was calculated as the sum of allowed amounts paid by Medicare, primary payer, co-insurers, and deductible payment during the year [27]. We evaluated *total medical spending* based on the MBSF cost and utilization claims (i.e., inpatient, outpatient, physician service, skilled nursing facility service, home health service, and other services; Supporting Information S2 Table shows the different categories of Medicare spending). Due to the high number of zero payments for the hospital and post-acute care categories (25.7% and 73.9%, respectively), only total spending and spending for physician services were presented. The amount of Medicare spending was winsorized to the 99th percentile to minimize the impact of outliers. For standardized cost comparison across years (2000–2013) and geographic areas, we first adjusted for inflation using the 2018 annual average of the consumer price index (CPI). Second, we standardized spending at the county level, using the 2007 CMS Geographic-Variation costs. This was done by dividing the standardized spending by the total actual spending in a given county (CMS Geographic-Variation costs). We then multiplied this ratio by the total medical spending. This ratio is fairly consistent across years [28].

*Medicare utilization*. We also calculated four dichotomous outcomes (zero vs. any use) to characterize health services utilization for each year. These categories included skilled nursing facility (SNF) use, home health use, hospitalization, and emergency room admission.

*High-cost beneficiaries*. We also examined the proportion of participants in each segment that were high cost. High-cost beneficiaries were defined as those with disproportionate share of spending, and if their total Medicare spending was in the highest 10% of total spending among all beneficiaries, in the same calendar year. This cut-off has been widely used in the literature to identify high-cost patients [7,14,29].

#### Sociodemographic and clinical characteristics

Beneficiaries’ characteristics included age (years), sex (female, male), years of education, marital status (married, not married), and dual eligibility for Medicare and Medicaid. Smoking and drinking alcohol were categorized as current/ever (yes, no). Cognitive impairment was assessed using the Mini-Mental State Examination (MMSE). Scores on the MMSE have a range of 0 to 30, with a score of 21 or greater indicating high cognitive function. Disability was assessed by using the activities of daily living (ADL) scale. ADL disability was dichotomized into no help needed vs needing help with–or unable to perform–one or more of the seven ADL activities. Frailty was assessed using the modified frailty phenotype measure [30], which includes weight loss, weakness, exhaustion, slowness, and limitations in walking half a mile. Low physical activity from original frailty phenotype was replaced by “walking half a mile” in the modified frailty phenotype measure. Participants were categorized as non-frail (0 criterion), pre-frail (1–2 criteria), or frail (3+ criteria) [31].

#### Social risk factors

We also included information regarding whether the beneficiary was living alone (by asking how many people live in the household), had financial strain (by asking whether it was difficult to pay bills), had someone to count on during times of trouble (most times, sometimes/hardly ever), had someone to talk about problems (most times, sometimes/hardly ever). These variables have been used as a proxy for family support [26].

#### Analysis

Using Chi-square test and Analysis of variance (ANOVA), we compared baseline sociodemographic and clinical characteristics among beneficiaries in the three population segments (major complex, minor complex and simple/healthy) to identify predictors of cost and utilization among these groups. We then estimated a series of regressions to examine differences in spending and utilization across the three segments. To evaluate differences in Medicare utilization and spending across the segments, we conducted a series of generalized estimating equation (GEE) models with first order autoregressive process, AR(1), as correlation matrix to adjust within subject dependence in the longitudinal data. GEE models were used because these data were longitudinal and correlated [32]. To evaluate differences in Medicare services utilization, we conducted a series of GEE models with a logit link binomial distribution to estimate the odds ratio (OR) for utilization. To evaluate differences in Medicare spending, a log link gamma distribution in GEE models was applied to estimate cost ratios (CR) of each of the population segments. Preliminary models were unadjusted while subsequent models were adjusted sequentially to examine the influence of frailty, sociodemographic, and clinical characteristics on spending and utilization across population segments. Model 2 adjusted for frailty, and Model 3 adjusted for age, sex, education, Medicaid, marital status, financial strain, cognitive function, and disability. Model 3 did not adjust for smoking/drinking alcohol, living alone, having someone to count on during times of trouble, and having someone to talk about problems due to high proportions of missing values or small cell sizes (See Table 1 and Supporting Information S3 Table). Lastly, we created a final model, in which we looked at the effect of frailty status among participants in the major complex chronic illness segment. All models included a year fixed-effect. Following prior studies that have used these data [25,33], we did not use sampling weights in this analysis. All analyses were conducted using SAS v. 9.4 (SAS Inc., Cary, NC).

**Table 1:** Baseline characteristics stratified by population segments (N = 863).

Variable	Total	Population Segments at Baseline			p-value
		Major complex	Minor complex	Simple/Healthy	
Number of Subject	N = 863	N = 229	N = 353	N = 281	
Age at beginning	78.3 (5.0)	79.2 (5.4)	78.3 (5.0)	77.7 (4.5)	0.0028
Sex					0.0090
Male	351 (40.7%)	86 (37.6%)	130 (36.8%)	135 (48.0%)	
Female	512 (59.3%)	143 (62.4%)	223 (63.2%)	146 (52.0%)	
Education, years^§^	4.8 (3.9)	4.5 (3.9)	5.0 (4.1)	5.0 (3.8)	0.3328
Marital status					0.2342
Married	394 (45.7%)	94 (41.0%)	164 (46.5%)	136 (48.4%)	
Not married	469 (54.3%)	135 (59.0%)	189 (53.5%)	145 (51.6%)	
Medicaid	609 (70.6%)	174 (76.0%)	257 (72.8%)	178 (63.3%)	0.0038
Live alone^§^	267 (31.0%)	76 (33.2%)	109 (31.1%)	82 (29.2%)	0.6228
Financial strain^§^					0.1468
Difficult to pay bill	514 (60.0%)	147 (64.8%)	197 (56.6%)	170 (60.5%)	
Little/None difficult	342 (40.0%)	80 (35.2%)	151 (43.4%)	111 (39.5%)	
Someone to count on^§^					0.5149
Most	696 (82.5%)	182 (83.1%)	289 (83.8%)	225 (80.4%)	
Some/Hardly	148 (17.5%)	37 (16.9%)	56 (16.2%)	55 (19.6%)	
Someone to talk^§^					0.2513
Most	669 (79.0%)	174 (78.7%)	282 (81.5%)	213 (76.1%)	
Some/Hardly	178 (21.0%)	47 (21.3%)	64 (18.5%)	67 (23.9%)	
Current Smoke/Drink^§^	167 (19.6%)	32 (14.3%)	61 (17.5%)	74 (26.4%)	0.0014
Cognitive function					
Total score	22.6 (5.3)	21.5 (5.8)	23.1 (5.3)	23.0 (4.7)	0.0005
MMSE≥21	581 (67.6%)	135 (59.2%)	251 (71.5%)	195 (69.4%)	0.0062
MMSE<21	279 (32.4%)	93 (40.8%)	100 (28.5%)	86 (30.6%)	
ADL^§^					<0.0001
No help	716 (83.1%)	157 (68.6%)	300 (85.0%)	259 (92.5%)	
Need help, disability	146 (16.9%)	72 (31.4%)	53 (15.0%)	21 (7.5%)	
Frailty					<0.0001
Robust	302 (35.0%)	55 (24.0%)	125 (35.4%)	122 (43.4%)	
Pre-Frail	458 (53.1%)	126 (55.0%)	187 (53.0%)	145 (51.6%)	
Frail	103 (11.9%)	48 (21.0%)	41 (11.6%)	14 (5.0%)	

^§^ There was missing data.

## Results

### Characteristics of population segments

Table 1 shows the baseline characteristics of the study population stratified by population segments. Beneficiaries in the major complex segment were older, more likely to be dual eligible for Medicare/Medicare, less likely to smoke or drink, more likely to have cognitive impairment, ADL disability, and were frail compared to those in the minor complex or simple/heathy segments (See Supporting Information S3 Table for a comparison regarding baseline characteristics among survey and study cohort).

### Medicare spending and utilization by population segments

Medical spending and utilization varied across the population segments in unadjusted models and when adjusting for frailty status (Table 2). In the unadjusted model, for the minor complex and major complex segments compared to the simple/healthy segment, there was a higher total cost (CR = 1.61, 95% CI, 1.29–2.01, and CR = 3.02, 95% CI, 2.46–3.71, respectively) and physician cost (CR = 1.52, 95% CI, 1.29–1.79 and CR = 2.51, 95% CI, 2.12–2.98, respectively). These two segments had higher use of home health, hospitalizations, and emergency room admissions. The major complex segment had the highest likelihood of being high cost (OR = 6.13, 95% CI, 3.63–10.38). These results remained fairly consistent after adjusting for frailty (see Model 2 in Table 3).

**Table 2:** Medicare spending and utilization by population segment.

Variable	Medicare Spending		Medicare Utilization				
	Total	Physician	High-Cost	SNF Use	Home Health Use	Hospitalization	ER admission
	CR (95%CI)	CR (95%CI)	OR (95%CI)	OR (95%CI)	OR (95%CI)	OR (95%CI)	OR (95%CI)
**Model 1**							
Minor	1.61 (1.29–2.01)*	1.52 (1.29–1.79)*	1.81 (1.03–3.20)*	2.11 (0.67–6.71)	1.99 (1.38–2.87)*	1.51 (1.09–2.11)*	1.77 (1.33–2.35)*
Major	3.02 (2.46–3.71)*	2.51 (2.12–2.98)*	6.13 (3.63–10.38)*	11.40 (3.98–32.64)*	6.01 (4.17–8.66)*	3.82 (2.76–5.29)*	4.02 (2.99–5.41)*
**Model 2**							
Minor	1.59 (1.28–1.99)*	1.51 (1.29–1.78)*	1.77 (1.01–3.13)*	2.05 (0.65–6.48)	1.98 (1.37–2.86)*	1.49 (1.07–2.07)*	1.73 (1.30–2.30)*
Major	2.90 (2.35–3.57)*	2.49 (2.10–2.95)*	5.76 (3.38–9.80)*	10.28 (3.65–28.94)*	5.75 (3.98–8.32)*	3.63 (2.62–5.04)*	3.79 (2.81–5.12)*
Pre-Frail	1.40 (1.19–1.66)*	1.20 (1.06–1.36)*	1.91 (1.24–2.93)*	1.49 (0.76–2.91)	1.67 (1.24–2.23)*	1.44 (1.10–1.88)*	1.29 (1.01–1.63)*
Frail	1.44 (1.15–1.81)*	1.10 (0.93–1.29)	1.83 (1.07–3.15)*	2.05 (0.96–4.37)	1.88 (1.30–2.72)*	1.54 (1.07–2.23)*	1.72 (1.22–2.44)*
**Model 3**							
Minor	1.67 (1.34–2.08)*	1.51 (1.28–1.77)*	1.79 (1.00–3.22)	2.08 (0.64–6.70)	2.03 (1.38–2.98)*	1.52 (1.09–2.12)*	1.77 (1.32–2.37)*
Major	2.88 (2.35–3.54)*	2.42 (2.04–2.87)*	5.51 (3.19–9.53)*	9.09 (3.18–26.01)*	5.59 (3.81–8.19)*	3.60 (2.57–5.04)*	3.91 (2.87–5.35)*
Pre-Frail	1.33 (1.11–1.59)*	1.17 (1.03–1.33)*	1.69 (1.06–2.68)*	1.13 (0.55–2.32)	1.51 (1.11–2.05)*	1.35 (1.02–1.79)*	1.22 (0.95–1.56)
Frail	1.26 (1.00–1.57)*	1.03 (0.87–1.22)	1.41 (0.77–2.60)	1.52 (0.63–3.67)	1.60 (1.06–2.41)*	1.44 (0.96–2.16)	1.57 (1.07–2.30)*
2006	1.37 (1.13–1.67)*	0.87 (0.76–1.00)	0.76 (0.48–1.21)	0.67 (0.32–1.41)	2.58 (1.86–3.58)*	0.93 (0.69–1.25)	0.77 (0.59–1.00)*
2008	1.39 (1.16–1.68)*	0.91 (0.78–1.05)	0.67 (0.40–1.11)	0.84 (0.42–1.66)	2.98 (2.06–4.31)*	0.96 (0.68–1.35)	0.69 (0.50–0.95)*
2012	1.20 (0.98–1.48)	0.90 (0.76–1.08)	0.59 (0.31–1.11)	0.78 (0.33–1.87)	2.63 (1.69–4.09)*	0.69 (0.44–1.07)	0.94 (0.65–1.38)
2014	1.26 (0.91–1.75)	0.95 (0.70–1.28)	0.54 (0.23–1.28)	0.46 (0.13–1.68)	2.38 (1.38–4.11)*	0.78 (0.45–1.35)	1.17 (0.72–1.88)
Age	0.99 (0.98–1.01)	0.99 (0.98–1.00)	1.00 (0.96–1.03)	1.04 (0.99–1.10)	1.01 (0.98–1.04)	1.02 (0.99–1.04)	1.03 (1.00–1.05)*
Medicaid	1.28 (1.08–1.53)*	1.16 (1.01–1.33)*	1.04 (0.67–1.61)	0.96 (0.47–1.99)	0.86 (0.60–1.23)	0.95 (0.72–1.26)	0.91 (0.68–1.22)
Not Married	1.05 (0.89–1.23)	1.01 (0.89–1.14)	0.81 (0.54–1.23)	1.92 (1.01–3.66)*	0.98 (0.72–1.34)	0.93 (0.71–1.23)	1.09 (0.84–1.42)
Financial strain	1.16 (0.99–1.36)	1.10 (0.98–1.23)	1.15 (0.79–1.68)	1.23 (0.71–2.13)	1.29 (0.98–1.71)	1.14 (0.88–1.48)	1.02 (0.81–1.29)
Disability	1.30 (1.11–1.53)*	1.18 (1.03–1.35)*	1.84 (1.20–2.83)*	1.32 (0.72–2.43)	1.67 (1.22–2.28)*	1.05 (0.77–1.42)	0.96 (0.73–1.27)

Notes: CI refers to confidence intervals; CR refers to cost ratios; SNF refers to skilled nursing facilities; ER refers to emergency room; Model 1: Outcome = calendar year fixed effect+ population segment; Model 2: Outcome = model 1 + Frailty; Model 3: Outcome = model 2 + age + sex + education + Medicaid + Marital status + financial strain + cognitive function (MMSE) + needing help with activities of daily living; Model 3 did not adjust for smoking/drinking alcohol, living alone, had someone to count on during times of trouble, and had someone to talk about problems due to high proportion of missing values or small cell sizes; * refers to significance at p<0.05.

**Table 3:** The impact of population segment combined with frailty on Medicare spending and utilization.

Variable	Medicare Spending		Medicare Utilization				
	Total	Physician	High-Cost	SNF Use	Home Health Use	Hospitalization	ER admission
	CR (95%CI)	CR (95%CI)	OR (95%CI)	OR (95%CI)	OR (95%CI)	OR (95%CI)	OR (95%CI)
Minor	1.68 (1.35–2.10)*	1.51 (1.28–1.78)*	1.81 (1.00–3.26)*	2.12 (0.66–6.82)	2.02 (1.38–2.96)*	1.54 (1.10–2.15)*	1.79 (1.34–2.40)*
Major without frail	2.59 (1.99–3.37)*	2.34 (1.88–2.91)*	4.92 (2.43–9.98)*	4.40 (1.03–18.75)*	5.00 (3.05–8.20)*	2.73 (1.71–4.36)*	3.58 (2.33–5.48)*
Major with pre-frail/frail	3.05 (2.48–3.75)*	2.45 (2.06–2.91)*	5.86 (3.35–10.25)*	11.32 (3.88–33.02)*	5.91 (3.96–8.82)*	4.12 (2.88–5.90)*	4.24 (3.04–5.91)*
2002 (reference)							
2006	1.37 (1.14–1.66)*	0.87 (0.76–1.00)*	0.77 (0.49–1.21)	0.69 (0.33–1.46)	2.62 (1.90–3.63)*	0.95 (0.70–1.28)	0.79 (0.61–1.02)
2008	1.38 (1.14–1.67)*	0.90 (0.78–1.05)	0.66 (0.39–1.09)	0.86 (0.43–1.73)	2.94 (2.04–4.24)*	0.96 (0.68–1.36)	0.70 (0.51–0.96)*
2012	1.18 (0.96–1.46)	0.90 (0.75–1.08)	0.59 (0.31–1.11)	0.79 (0.33–1.86)	2.64 (1.70–4.11)*	0.69 (0.44–1.07)	0.96 (0.66–1.40)
2014	1.30 (0.94–1.81)	0.96 (0.71–1.30)	0.56 (0.23–1.33)	0.47 (0.13–1.66)	2.49 (1.44–4.31)*	0.81 (0.47–1.39)	1.24 (0.78–1.98)
Age	0.99 (0.98–1.01)	0.99 (0.98–1.00)	1.00 (0.97–1.04)	1.04 (0.98–1.10)	1.02 (0.98–1.05)	1.02 (0.99–1.05)	1.03 (1.01–1.06)*
Female	0.95 (0.79–1.14)	1.03 (0.90–1.18)	0.94 (0.62–1.43)	1.08 (0.57–2.06)	1.35 (0.98–1.86)	1.10 (0.83–1.47)	1.31 (1.00–1.72)*
Medicaid	1.30 (1.09–1.54)*	1.17 (1.02–1.34)*	1.07 (0.69–1.65)	0.95 (0.46–1.96)	0.89 (0.62–1.26)	0.97 (0.73–1.28)	0.93 (0.69–1.24)
Financial strain	1.19 (1.02–1.38)*	1.11 (0.99–1.24)	1.18 (0.81–1.72)	1.22 (0.69–2.13)	1.32 (1.00–1.74)	1.15 (0.89–1.49)	1.03 (0.82–1.30)
Disability	1.33 (1.12–1.57)*	1.17 (1.03–1.34)*	1.88 (1.23–2.87)*	1.33 (0.75–2.35)	1.76 (1.30–2.38)*	1.07 (0.79–1.44)	1.03 (0.79–1.35)

Notes: CI refers to confidence intervals; CR refers to cost ratios; Model: Outcome = Calendar year effect + Segment combined with frailty status + age + sex + education + Medicaid + Marital status + financial strain + cognitive function (MMSE) + needing help with activities of daily living; This model did not adjust for smoking/drinking alcohol, living alone, had someone to count on during times of trouble, and had someone to talk about problems due to high proportion of missing values or small cell sizes; * p<0.05.

Results from Model 3 show similar patterns of the impact of these population segments on Medicare spending and utilization. The CRs for total Medicare spending were 1.67 (95% CI, 1.34–2.08) and 2.88 (95% CI, 2.35–3.54) for minor and major complex patients compared to simple/healthy patients and 1.26 (95% CI, 1.00–1.57) for frail compared to non-frail. In terms of Medicare utilization, complex patients still were more likely to be considered high-need 5.51 OR (95% CI, 3.19–9.53) and had higher ORs of SNF care 9.09 (95% CI, 3.18–26.01), home health 5.59 (95% CI, 3.81–8.19) and emergency room admissions 3.91 (95% CI, 2.87–5.35) compared to simple/healthy patients.

#### Major Medicare spending and utilization compounded by frailty status

After accounting for covariates, the results show that, compared to the simple/healthy segment, the major chronic conditions with pre-frail/frail have higher total Medicare costs and utilization in all categories (See Table 3). The CR for total Medicare spending was 3.05 (95% CI, 2.48–3.75). Similarly, this group had higher odds of being classified as high cost 5.86 (95% CI, 3.35–10.25), have SNF care utilization 11.32 (95% CI, 3.88–33.02), home health 5.91 (95% CI, 3.96–8.82), hospitalization 4.12 (95% CI, 2.88–5.90) and emergency room admissions 4.24 (95% CI, 3.04–5.91).

## Discussion

In this study of Mexican Americans Medicare beneficiaries, we found that those with major complex chronic illness had much higher odds of being high cost, and these odds were slightly higher when major conditions were paired with frailty status. Beneficiaries in the major and minor complex illness segments had about two times greater total Medicare spending and greater health care utilization when compared to healthy beneficiaries. Another key finding is the relatively high percentage of beneficiaries who were dually eligible for Medicaid/Medicare and who had cognitive impairment/disability. About 70% of all these beneficiaries were dually eligible, which means that even among those relatively healthy, 63% were eligible for Medicare and Medicaid. Similarly, approximately two-thirds of all beneficiaries were classified as pre-frail or frail, and about one-third of all beneficiaries had low cognitive function. Finally, hospitalizations followed by other services (e.g., hospice, dialysis, imaging, tests) were the two major drivers of Medicare costs among Mexican American population segments. At baseline, hospitalizations, other services, and post-acute care accounted for about 60%, 18% and 15% of total Medicare spending among high-need beneficiaries, respectively (See S4 Table in the Supporting Information).

Our results are consistent with prior literature that suggests that frailty is an important indicator of higher Medicare spending. Prior research revealed that high-cost frail adults account for 44% of preventable Medicare spending (2012 US $6,593 per beneficiary) [5]. The original segmentation strategy adapted in the present study included frailty as a separate category [14]. However, frailty prevalence increases in older adults as a function of the accumulation of chronic conditions [15]. Therefore, conceptually and clinically, frailty status should not be considered to be a mutually exclusive category among medically complex older adults. This may be relevant because we found evidence of frailty across all segments—5% of those relatively healthy, 12% of minor complex and 21% of major complex. Also consistent with prior work, we found that HNHC Mexican American older adults have functional limitations and a combination of multiple chronic conditions [3,6,34].

Similarly, others have found that high-cost patients were more likely to be enrolled in Medicare and Medicaid and have financial strains [3]. Our sample included high rates of dual eligible beneficiaries, with nearly double the rate compared to other studies with more diverse populations [29]. Dual eligible beneficiaries have to navigate state and federal policies that were not properly designed to work together [35]. Medicare primarily covers inpatient hospitalization, post-acute care and some prescription drugs. Medicaid primarily pays for long-term care services and supports, some behavioral health services and Medicare cost-sharing for dual eligible beneficiaries [36]. Medicare spending on dual eligible beneficiaries is higher than non-duals due to their complex health care needs [37]. Dual eligible beneficiaries have a high prevalence of chronic conditions (e.g., diabetes, heart failure, hypertension and Alzheimer’s disease) [37]. In fact, about 60% of dual eligible beneficiaries have two or more chronic conditions, which increases the use of specific services (e.g., inpatient hospital, post-acute care services, outpatient services, and Part D drugs) [36,37]. Our results show higher Medicare spending among dual eligible beneficiaries.

Our study results have major policy implications. Some studies have identified Hispanic older adults and dual eligible beneficiaries as being high-cost [29,38]. However, classifying individuals in such studies rely on the use of administrative and post-acute assessment claims. Therefore, utilization of services is an integral component of being identified as HNHC [34]. Interestingly, others have found that Hispanic beneficiaries appear to have the lowest average annual per beneficiary spending for dual eligible beneficiaries and non-dual eligible beneficiaries when compared to other racial and ethnic groups. In 2015, spending among dual eligible beneficiaries and non-dual eligible beneficiaries was $11,438 and $4,096 for Hispanics, $16,591 and $6,694 for African Americans, and $14,919 and $6,893 for White beneficiaries [39]. We found a large proportion of Mexican American older adults with characteristics associated with high-need status, such as frailty, functional limitations, and cognitive impairment, who would not be categorized as being HNHC. Systematic assessment of function, frailty, and cognitive screening in primary care settings may assist health professionals and providers to identify individuals earlier in the continuum of care, which may allow for tailored interventions to deter excessive or unwarranted emergency department visits, hospitalizations, and readmissions.

Work by the Medicaid and CHIP Payment and Access Commission (MACPAC) has shown that lack of coordination in Medicare and Medicaid services contributes to fragmented care and poor outcomes among dually eligible beneficiaries [35]. Therefore, federal and state governments have been implementing different strategies and alternative payment models to improve Medicare-Medicaid care coordination and enhance access to quality services while containing costs for dually eligible beneficiaries [36]. One of these key changes is aimed at reducing improper billing to dual beneficiaries [40]. However, there is a push to coordinate and provide benefits through Medicare-Medicaid Plans (MMPs) and especially through Medicare Advantage Dual eligible special needs plans (D-SNPs) [41,42]. These programs are targeted to the specific needs of dually eligible beneficiaries and integrate different health services (i.e., primary, acute, and behavioral health care, and long-term services and supports) and/or social determinants of health [41]. Particularly, Medicare Advantage plans may offer services or work with local programs to deal with issues related to food insecurity, transportation, technology literacy, social isolation/loneliness, housing instability and homelessness [41,43]. Thus, the proportion of beneficiaries in these plans has increased exponentially in recent years, with high rates of enrollment among Hispanics and other minority groups [44].

As of 2021, about 26% (~ 3 million) of the dually eligible population were enrolled in D-SNPs [42]. Given the rapid growth of D-SNPs among Hispanics [44]—and potentially among Mexican Americans—determining whether managed care programs are effective in improving health quality and outcomes in this population should be a priority for federal and local governments. This is particularly challenging now because claims and cost data are not available for enrollees under managed care.

Similarly, a greater understanding of factors related to being HNHC is needed to improve outcomes and reduce spending among dual eligible beneficiaries. These factors are particularly important when considering how to identify individuals who may be HNHC in Hispanic populations using proposed definitions, as traditionally post-acute care and nursing home care utilization has been low among Hispanic older adults, which may underestimate the proportion of HNHC for this population [45]. Although this trend may be expected to change in the future, it is unclear how disproportionate death rates related to COVID-19 will influence post-acute and nursing home utilization among this population. What is known from the research [46–48] is that better access to quality providers is needed among Hispanic and other minority groups. Hispanics often received post-acute or long-term care in more segregated nursing homes with fewer resources and lower quality of care including higher readmission rates and lower star ratings, compared with White residents [46,48]. It is expected that this may be similar among the small proportion of Hispanics with access to assisted living facilities. Unfortunately, this is not exclusive to long-term care services and support. Inequities in access and quality of care are prevalent across a variety of healthcare settings [49]. Therefore, it is important to identify proper interventions to improve care and reduce fragmented care and unnecessary expenses across the healthcare system.

Our results have several limitations. First, we use Medicare administrative data to capture the number of chronic conditions. Therefore, we lacked information about the severity of chronic illnesses, which could impact the population segments. However, we included survey information related to ADLs, cognitive function, and frailty. In addition, there were 2,580 H-EPESE respondents linked to the Medicare data. However, only 863 unique individuals have complete information related to chronic conditions, frailty assessments, healthcare cost, and utilization. A higher proportion of individuals included in the cohort were younger, had high cognitive function, did not need help with ADLs, and were classified as non-frail (See S3 Table in the Supporting Information). Therefore, cost and utilization may be underestimated for this population.

Second, although we classified Medicare cost and utilization using categories that others have suggested, due to sample size we were unable to identify unavoidable and preventable hospitalizations. We were also unable to provide information regarding beneficiaries that are persistently high-cost or transiently high-cost. Third, dual eligibility for Medicare and Medicaid included both partially and fully eligible for Medicaid. Similarly, health care cost and utilization came from the Master Beneficiary Summary File (MBSF): Cost and Utilization Segment (See S2 Table in the Supporting Information for a full description of services included in the study). This is based on Medicare spending, which may have underestimated non-institutional health services utilization and the costs of dual-eligible beneficiaries. For all enrollees in the present study (including dual beneficiaries), we are only calculating costs related to Medicare Part A and Part B. Medicare Part A covers inpatient care in acute hospitals, inpatient rehabilitation, and skilled nursing facility (≥100 days), hospice, and home health care; Medicare Part B covers doctor and other health care providers’ services, outpatient care, durable medical equipment, and some preventive services; Medicare covers care in a SNF up to 100 days and after that custodial and/or long-term care services are covered by Medicaid or out-of-pocket [50]. We do not have health care costs and/or utilization related to long-term care services and supports, and Medicare premiums and/or cost-sharing which are often covered by Medicaid [36]. Thus, per beneficiary spending may be underestimated. About 50% of dual beneficiaries use long-term services and support [36]. These services account for about 70% of the total spending among all high-cost duals ($161, 224) [38]. However, this may not be the case among our study population, who were community dwelling. Similarly, another study found that any nursing home care spending levels and growth were higher among non-dual beneficiaries [39]. Thus, data from the present study may not reflect current patterns of spending and utilization, specifically related to long-term care services and utilization and/or post-acute care. However, we provide information about social burden and support among these beneficiaries. Finally, we did not include data related to nativity status such as being U.S.- or foreign-born. However, a prior study found no differences in terms of health services utilization (hospitalizations, ER admissions and outpatient visits) among U.S.- or foreign-born Mexican Americans enrolled in Medicare [25].

## Conclusions

Our findings highlight the importance of assessing healthcare cost and utilization among Mexican Americans. It also underscores the need to use and incorporate frailty assessment when identifying HNHC patients. Understanding differences in health care utilization among high-cost population segments may assist healthcare providers to develop interventions to improve care delivery, reduce expenditures, and improve quality of life and outcomes for Mexican American older adults.

## Supporting information

S1 Table Classification based on the chronic conditions in the master beneficiary summary file. Joynt and colleagues [14] defined 29 chronic conditions category (9 complex and 20 others) based on CMS-HCC and CCW (https://www2.ccwdata.org/web/guest/condition-categories). For our study population, CCW flag could cover 25 categories (9 complex and 17 others) and survey questionnaire could cover 17 categories (7 complex and 10 others). People in this sample did not have immune disorder; inflammatory bowel disease; neuromuscular disease; paralytic diseases; skin ulcer; or substance abuse.
(DOCX)

S2 Table The element of each subcategory on Medicare spending. MBSF: Master Beneficiary Summary File.
(DOCX)

S3 Table Baseline characteristics among survey cohort, linkage cohort, and study cohort§. § Wave 1 and wave 5 interview assessments were presented as baseline characteristics for subjects recruiting in 1993/1994 and in 2004/2005 separately. † Chi-Square goodness of fit test between survey and linkage population (p<0.01). ‡ Chi-Square goodness of fit test between survey and study population (p<0.01).
(DOCX)

S4 Table Percent of spending for each category stratified by baseline high-need status. Note: There were 48 subjects from not high-need group with 0 spending at baseline and they were not included in the analysis; Wilcoxon rank-rum test was applied to compare the median percent of spending between two groups; Hospital service spending (%) = Hospital service spending/Total spending * 100%; Physician service spending (%) = Physician service spending/Total spending * 100%; Post-acute care spending (%) = Post-acute care spending/Total spending * 100%; Other service spending (%) = Other service spending/Total spending * 100%.
(DOCX)
