# Supplementary material for: Healthcare utilization and costs among high-need and frail Mexican American Medicare beneficiaries
Source: PLoS One. 2022 Jan 14;17(1):e0262079. doi: 10.1371/journal.pone.0262079 (PMC8759642; doi:10.1371/journal.pone.0262079)
Supplement: S1 Table — Joynt and colleagues [14] defined 29 chronic conditions category (9 complex and 20 others) based on CMS-HCC and CCW (https://www2.ccwdata.org/web/guest/condition-categories). For our study population, CCW flag could cover 25 categories (9 complex and 17 others) and survey questionnaire could cover 17 categories (7 complex and 10 others). People in this sample did not have immune disorder; inflammatory bowel disease; neuromuscular disease; paralytic diseases; skin ulcer; or substance abuse. (DOCX) [file pone.0262079.s001.docx]

**S1 Table. Classification Based on the Chronic Conditions in the Master Beneficiary Summary File**

| **Complex Chronic Conditions** |
| --- |
| **Acute MI/Ischemic Heart Disease**  Acute Myocardial Infarction; Ischemic Heart Disease (either one) |
| **Chronic Kidney Disease**  Chronic Kidney Disease |
| **Congestive Heart Failure**  Heart Failure |
| **Diabetes**  Diabetes |
| **Dementia**  Alzheimer's Disease, Related Disorders, or Senile Dementia |
| **Lung Disease**  Chronic Obstructive Pulmonary Disease |
| **Psychiatric Disease**  Schizophrenia; Depression; Bipolar Disorder; Personality Disorders (either one) |
| **Specified Heart Arrhythmias**  Atrial Fibrillation |
| **Stroke**  Stroke / Transient Ischemic Attack |
| **Other Chronic Conditions** |
| **Amputation status**  Hip/Pelvic Fracture; Mobility Impairments (either one) |
| **Arthritis and Other Inflammatory Tissue Disease**  Rheumatoid Arthritis / Osteoarthritis |
| **Benign Prostatic Hyperplasia**  Benign Prostatic Hyperplasia |
| **Cancer**  Colorectal, Endometrial, Breast, Lung, and Prostate cancer; Leukemia and Lymphomas (either one) |
| **Cystic Fibrosis**  Cystic Fibrosis and Other Metabolic Developmental Disorders |
| **Endocrine And Metabolic Disorders**  Obesity |
| **Eye Disease**  Cataract; Glaucoma (either one) |
| **Hematological Disease**  Anemia |
| **Hyperlipidemia**  Hyperlipidemia |
| **Hypertension**  Hypertension |

**S1 Table (continued)**

| **Other Chronic Conditions** |
| --- |
| **Liver & Biliary Disease**  Liver Disease, Cirrhosis and Other Liver Conditions |
| **Neuromuscular Disease**  Multiple Sclerosis and Transverse Myelitis; Muscular Dystrophy (either one) |
| **Osteoporosis**  Osteoporosis |
| **Paralytic Diseases / Conditions**  Cerebral Palsy |
| **Skin Ulcer**  Pressure and Chronic Ulcers |
| **Substance Abuse**  Drug Use Disorders |
| **Thyroid Disease**  Acquired Hypothyroidism |

Note: Joynt et al. defined 29 chronic conditions category (9 complex and 20 others) based on CMS-HCC and CCW (<https://www2.ccwdata.org/web/guest/condition-categories>). For our study population, CCW flag could cover 25 categories (9 complex and 17 others) and survey questionnaire could cover 17 categories (7 complex and 10 others). People in this sample did not have immune disorder; inflammatory bowel disease; neuromuscular disease; paralytic diseases; skin ulcer; or substance abuse.
