# Supplementary material for: Healthcare utilization and costs among high-need and frail Mexican American Medicare beneficiaries
Source: PLoS One. 2022 Jan 14;17(1):e0262079. doi: 10.1371/journal.pone.0262079 (PMC8759642; doi:10.1371/journal.pone.0262079)
Supplement: S2 Table — MBSF: Master Beneficiary Summary File. (DOCX) [file pone.0262079.s002.docx]

**S2 Table. The Element of Each Subcategory on Medicare Spending**

| CATEGORY | TYPE OF CLAIMS IN MBSF COST AND UTILIZATION SEGMENT |
| --- | --- |
| Hospital Service (Inpatient) | Acute Inpatient  Other Inpatient |
| Hospital Service (Outpatient) | Hospital Outpatient |
| Physician Service | Evaluation and Management  Part B Physician |
| SNF Service | Skilled Nursing Facility |
| Home Health Service | Home Health |
| Other Service | Ambulatory Surgery Center  Hospice  Dialysis  Other Procedures  Imaging  Tests  Durable Medical Equipment  Part B  Anesthesia  Other Part B Carrier  Part D |

Note: MBSF: Master Beneficiary Summary File
