# Supplementary material for: Healthcare utilization and costs among high-need and frail Mexican American Medicare beneficiaries
Source: PLoS One. 2022 Jan 14;17(1):e0262079. doi: 10.1371/journal.pone.0262079 (PMC8759642; doi:10.1371/journal.pone.0262079)
Supplement: S3 Table — § Wave 1 and wave 5 interview assessments were presented as baseline characteristics for subjects recruiting in 1993/1994 and in 2004/2005 separately. † Chi-Square goodness of fit test between survey and linkage population (p<0.01). ‡ Chi-Square goodness of fit test between survey and study population (p<0.01). (DOCX) [file pone.0262079.s003.docx]

**S3 Table. Baseline Characteristics among Survey Cohort, Linkage Cohort, and Study Cohort§**

| Variable | Survey | Linkage | Study |
| --- | --- | --- | --- |
|  | N=3952 | N=2580 | N=863 |
| Age in 2000^‡^ |  |  |  |
| <80 years | 2576 (65.2%) | 1730 (67.1%) | 657 (76.1%) |
| ≥80 years | 1373 (34.8%) | 850 (32.9%) | 206 (23.9%) |
| Sex |  |  |  |
| Male | 1662 (42.1%) | 1103 (42.8%) | 351 (40.7%) |
| Female | 2290 (57.9%) | 1477 (57.2%) | 512 (59.3%) |
| Education |  |  |  |
| <5 years | 2065 (57.4%) | 1318 (55.8%) | 481 (60.7%) |
| ≥6 years | 1534 (42.6%) | 1046 (44.2%) | 311 (39.3%) |
| Marital status^‡^ |  |  |  |
| Married | 2096 (53.1%) | 1422 (55.2%) | 514 (59.7%) |
| Not married | 1853 (46.9%) | 1156 (44.8%) | 347 (40.3%) |
| Live arrangement |  |  |  |
| With someone | 3045 (77.0%) | 1968 (76.3%) | 679 (78.7%) |
| Alone | 907 (23.0%) | 612 (23.7%) | 184 (21.3%) |
| Financial strain^‡^ |  |  |  |
| Difficult to pay bill | 2102 (58.9%) | 1404 (58.3%) | 537 (64.4%) |
| Little/None difficult | 1464 (41.1%) | 1006 (41.7%) | 297 (35.6%) |
| Someone to count on |  |  |  |
| Most | 2730 (76.3%) | 1839 (76.6%) | 640 (77.2%) |
| Some/Hardly | 849 (23.7%) | 561 (23.4%) | 189 (22.8%) |
| Someone to talk |  |  |  |
| Most | 2617 (73.2%) | 1774 (73.9%) | 633 (76.3%) |
| Some/Hardly | 960 (26.8%) | 627 (26.1%) | 197 (23.7%) |
| Current Smoke/Drink^†‡^ |  |  |  |
| Yes | 892 (38.4%) | 627 (42.5%) | 234 (50.6%) |
| No | 1430 (61.6%) | 848 (57.5%) | 228 (49.4%) |
| Cognitive function^†‡^ |  |  |  |
| Normal (MMSE≥21) | 2902 (78.2%) | 2019 (81.9%) | 756 (90.0%) |
| Impairment (MMSE<21) | 809 (21.8%) | 445 (18.1%) | 84 (10.0%) |
| ADL^†‡^ |  |  |  |
| No help | 3189 (80.9%) | 2177 (84.5%) | 811 (94.1%) |
| Need help, disability | 753 (19.1%) | 399 (15.5%) | 51 (5.9%) |
| Frailty^‡^ |  |  |  |
| Non-Frail | 998 (44.9%) | 788 (47.1%) | 387 (52.7%) |
| Pre-Frail | 1009 (45.3%) | 741 (44.3%) | 316 (43.0%) |
| Frail | 218 (9.8%) | 145 (8.7%) | 32 (4.4%) |

Note: § Wave 1 and wave 5 interview assessments were presented as baseline characteristics for subjects recruiting in 1993/1994 and in 2004/2005 separately.

† Chi-Square goodness of fit test between survey and linkage population (p<0.01)

‡ Chi-Square goodness of fit test between survey and study population (p<0.01)
