# Supplementary material for: Healthcare utilization and costs among high-need and frail Mexican American Medicare beneficiaries
Source: PLoS One. 2022 Jan 14;17(1):e0262079. doi: 10.1371/journal.pone.0262079 (PMC8759642; doi:10.1371/journal.pone.0262079)
Supplement: S4 Table — Note: There were 48 subjects from not high-need group with 0 spending at baseline and they were not included in the analysis; Wilcoxon rank-rum test was applied to compare the median percent of spending between two groups; Hospital service spending (%) = Hospital service spending/Total spending * 100%; Physician service spending (%) = Physician service spending/Total spending * 100%; Post-acute care spending (%) = Post-acute care spending/Total spending * 100%; Other service spending (%) = Other service spending/Total spending * 100%. (DOCX) [file pone.0262079.s004.docx]

**S4 Table. Percent of Spending for Each Category Stratified by Baseline High-Need Status**

| Category | High-Need | Not High-Need | p-value |
| --- | --- | --- | --- |
|  | N=83 | N=732 |  |
| Hospital service, % |  |  | <0.001 |
| Mean (SD) | 59.33 (19.48) | 26.42 (27.29) |  |
| Median (Q1-Q3) | 61.43 (45.41-75.61) | 16.16 (0.71-47.59) |  |
| Physician service, % |  |  | <0.001 |
| Mean (SD) | 7.21 (3.01) | 28.78 (24.98) |  |
| Median (Q1-Q3) | 6.78 (5.53-8.98) | 20.49 (9.66-41.52) |  |
| Post-acute care, % |  |  | <0.001 |
| Mean (SD) | 15.25 (16.51) | 4.84 (15.02) |  |
| Median (Q1-Q3) | 9.95 (0.00-25.95) | 0.00 (0.00-0.00) |  |
| Other service, % |  |  | <0.001 |
| Mean (SD) | 18.20 (11.26) | 39.95 (26.95) |  |
| Median (Q1-Q3) | 14.81 (9.85-24.32) | 35.46 (18.12-61.40) |  |

Note: there were 48 subjects from not high-need group with 0 spending at baseline and they were not included in the analysis; Wilcoxon rank-rum test was applied to compare the median percent of spending between two groups; Hospital service spending (%) = Hospital service spending / Total spending * 100% ; Physician service spending (%) = Physician service spending / Total spending * 100%; Post-acute care spending (%) = Post-acute care spending / Total spending * 100%; Other service spending (%) = Other service spending / Total spending * 100%
